# Has Glioblastoma Five- and Ten-Year Survival Been Affected by New Treatment Algorithms in Population-Based Data? A Retrospective Swedish Survey Between 1958 and 2015

**DOI:** 10.3390/cancers18111811

**Published:** 2026-06-01

**Authors:** Peter Milos, Erik Ehinger, Edward Visse, Peter Siesjö

**Affiliations:** 1 Department of Biomedical and Clinical Sciences, Linköping University, 581 83 Linköping, Sweden; peter.milos@regionostergotland.se; 2 Department of Neurosurgery, Linköping University Hospital, 581 85 Linköping, Sweden; 3 Neurosurgery, Department of Clinical Sciences Lund, Lund University, 221 84 Lund, Sweden; erik.ehinger@med.lu.se (E.E.); edward.visse@med.lu.se (E.V.); 4 Department of Neurosurgery, Skåne University Hospital, 221 85 Lund, Sweden

**Keywords:** glioblastoma, Sweden, five-year survival, ten-year survival, temozolomide

## Abstract

Background: Glioblastoma carries a dismal prognosis, with only a small proportion of patients surviving beyond five or ten years. Although the introduction of new oncological therapies has improved median overall survival among treated patients, their impact on long-term survival in population-based cohorts remains unclear. The primary aim of this study was to analyze temporal trends in long-term survival in Sweden. Methods: Long-term survivors with glioblastoma were analyzed in two cohorts. The first cohort comprised patients from all of Sweden identified through the Swedish Cancer Registry and the Cause of Death Registry between 1958 and 1999. The second cohort consisted of patients from Southeastern Sweden identified through the Swedish Brain Tumor Registry between 2000 and 2015. Results: Following a review of the original pathology reports, the diagnosis of glioblastoma among long-term survivors was found to be incorrect in 88% and 40% of cases in the first and second cohorts, respectively. In total, 89 five-year survivors were identified, including 66 and 23 patients in the respective cohorts. Younger age (*p* = 0.001) and good performance status (*p* = 0.014), but not sex, recurrence, or extent of resection, were significantly associated with five-year survival. Thirty-six patients (40%) survived for more than ten years. A significant gradual increase in five-year survival was observed following the introduction of the Stupp regimen in 2005, rising from 1.0% in 1990–1999 to 1.7% in 2000–2004, 3.8% in 2005–2009, and 4.7% in 2010–2015 (*p* = 0.007). In contrast, ten-year survival remained approximately 1% and did not increase over time. Conclusions: Five-year survival in glioblastoma increased significantly in Southeastern Sweden after 2005, possibly reflecting the impact of modern neuro-oncological treatment strategies. However, ten-year survival remained unchanged. A small subset of glioblastoma patients can nevertheless achieve very long survival and, in rare cases, complete remission.

## Simple Summary

Although median and short-term overall survival in patients with glioblastoma has improved somewhat in recent decades, 5- and 10-year survival remain rare. Whether long-term survival in glioblastoma has changed over time following the introduction of new treatment algorithms remains an unresolved question, particularly in population-based settings. In this study, we analyzed long-term survival in glioblastoma using tumor registry data spanning more than five decades in Sweden and Southeastern Sweden. We found a significant increase in 5-year survival, but not in 10-year survival, following the introduction of the Stupp regimen in 2005. Nevertheless, a small subset of glioblastoma patients experiences very long survival and, in rare cases, complete remission of the disease.

## 1. Introduction

Glioblastoma multiforme (GBM) is the most common malignant primary brain tumor and carries one of the worst prognoses among all human malignancies, with shorter median survival and lower five-year survival rates than both pancreatic cancer and mesothelioma [1]. GBM is characterized by aggressive growth and extensive dissemination of tumor cells beyond the main tumor mass, infiltrating eloquent brain regions and thereby limiting the possibility of radical surgical resection [2,3,4]. The biology of GBM is highly complex, with substantial genetic and molecular heterogeneity both between patients and within individual tumors, making targeted oncological treatment particularly challenging [5,6].

The current standard of care consists of maximal safe surgical resection followed by radiotherapy with concomitant temozolomide and adjuvant temozolomide chemotherapy [7]. Median survival among patients receiving this regimen is approximately 14 months [8]. However, because not all patients are eligible for or receive standard treatment, population-based median overall survival (OS) for all diagnosed GBM patients is considerably lower, ranging from 6 to 12 months [9,10,11,12]. Most patients still die within two years of diagnosis [13]. Nevertheless, a minority of patients survive substantially longer and are commonly referred to as long-term survivors (LTSs). The definition of LTS is somewhat inconsistent in the literature, ranging from 3- to 5-year survival, although five- and ten-year survival are commonly used benchmarks for human malignancies [14,15]. Reported five-year survival rates for GBM have historically varied between 1% and 19%, depending on population sampling, selection methods, study design, diagnostic accuracy, geographical region, and treatment strategies [16,17,18,19,20]. More recent population-based data estimate five-year survival to be approximately 7.0% across all age groups [10]. Very long-term survival (VLTS), often defined as survival of ≥10 years, is exceedingly rare, reported at approximately 0.7% in a meta-analysis [21], although rates as high as 4.4% for all ages have been described in the most recent CBTRUS report [10]. There have also been reports suggesting the existence of a “cure fraction” among GBM patients, with some individuals ultimately dying from causes unrelated to their tumor [22].

Several studies have investigated clinical and molecular characteristics associated with LTS by comparing long-term survivors (LTSs) with short-term survivors (STSs). Younger age, good performance status, and MGMT promoter methylation are among the most frequently reported, although not universally consistent, factors associated with prolonged survival [23,24,25,26,27]. Other proposed prognostic factors include a favorable immunological tumor microenvironment characterized by increased cytotoxic CD8+ T-cell infiltration and reduced regulatory T-cell (Treg)-mediated immunosuppression [28]. However, studies have thus far failed to identify universal and unbiased molecular prognostic markers, suggesting that LTS in GBM is likely multifactorial in origin [29]. Exceptional responses to neuro-oncological therapy due to genetic, epigenetic, or other biological mechanisms, as well as substantial biological heterogeneity within tumors classified as glioblastoma, have also been proposed as explanations for prolonged survival [30].

Several investigators have reported an overall improvement in GBM survival following the introduction of the Stupp regimen in 2005 [31,32,33]. To adequately address this issue while minimizing selection bias, analyses based on population-level data extracted from tumor registries are essential [34].

Currently, approximately 400 patients are diagnosed with GBM annually in Sweden, corresponding to an incidence of about 4 per 100,000 inhabitants per year [35]. The primary aim of the present study was therefore to analyze temporal trends in five- and ten-year survival among GBM patients using population-based registry data from Sweden between 1958 and 2015.

## 2. Material and Methods

Since the aim of this study was to investigate long-term patient- and treatment-related effects on five- and ten-year survival in GBM using registry-based data from Sweden and the Southeastern region of Sweden, patients diagnosed with GBM up to 2015 were included. The final follow-up date was 31 December 2025.

### 2.1. Patient Identification

Data were extracted from the Swedish Cancer Registry (SCR) for the period 1958–1999 using topography codes 193.0 (ICD-7), 191.9 (ICD-9), and C71.0–9 (ICD-10), together with morphology codes 476 (before ICD-O) and 93803, 94403, 94013, 94413, and 94423 (ICD-O after 1990). Data from the Swedish Cause of Death Register (SCDR) were also used to identify five-year survivors across all age groups.

Additional data were extracted from the Swedish Brain Tumor Registry (SBTR) for adult patients (>18 years) in the Southeastern region of Sweden between 2000 and 2015, using topography codes ICD-10 C71.0–9 and morphology codes 476, 93803, 94403, 94013, 94413, and 94423. The Southeastern healthcare region constitutes one of six healthcare regions in Sweden and serves a population of approximately one million inhabitants.

The SCR was established in 1958 and includes all cancer diagnoses in Sweden. The registry has been evaluated with an overall completeness of approximately 96–98% [36,37]. However, one review noted a tendency toward underreporting of nervous system tumors, particularly among patients older than 70 years [37].

The SBTR was initiated in 1999 and gradually replaced the SCR as the primary population-based registry for brain tumor patients. Registry coverage in the Southeastern region of Sweden has been reported to be nearly complete, with a registration frequency of 98–100% [38]. Despite this high level of coverage, concerns have been raised regarding the quality of some registered data, related both to registration procedures and subsequent data handling.

### 2.2. Patient, Symptom, Pathology, and Treatment Characteristics

The full pathology reports, neurosurgical records, and oncological charts of all retrievable GBM five-year survivors were reviewed by the authors. Only patients with histopathological findings consistent with primary GBM—defined by the presence of mitotic activity, hypercellularity, vascular proliferation, and necrosis according to the 2007 WHO Classification of Tumours of the Central Nervous System—were included. Patients with evidence of previous lower-grade glioma (LGG), oligodendroglioma, or oligoastrocytoma were excluded. No additional histopathological re-evaluation of tissue samples was performed.

Clinical data extracted from verified GBM five-year survivors included previous medical conditions, presenting symptoms, functional status, history of trauma or infection, prior tumors, radiological findings, neurosurgical procedures, reoperations and recurrence, oncological treatments, and overall survival. Extent of resection was categorized as gross total resection (GTR), partial resection (PR), or biopsy. Because postoperative MRI was not routinely performed in the Southeastern region before approximately 2008, the extent of resection in earlier cases was estimated based on operative reports. Apart from preoperative performance status, postoperative neurological complications, quality of life, and return-to-work status during stable disease were not investigated in detail.

All five-year survivors received standard-of-care treatment according to the treatment protocols applicable during their respective time periods. No patients were enrolled in clinical trials or received experimental therapies.

The annual incidence of GBM has been approximately 400 cases in Sweden overall and approximately 40 cases in the Southeastern region. Postoperative treatment according to the Stupp regimen was introduced as standard therapy around 2005, and fluorescence-guided resection was implemented around 2007.

### 2.3. Statistical Analysis

Descriptive statistics, tables, and figures were used to summarize clinical and epidemiological data. The chi-square test was applied to compare categorical variables between different time periods. Overall survival (OS) was defined as the time from the first surgical intervention to death. Differences in OS between age groups and time periods were analyzed using the log-rank test and illustrated with Kaplan–Meier curves. Patients without an event were censored at the date of the last available follow-up before statistical analysis.

Univariate analyses were performed using log-rank tests, Cox regression, and logistic regression. Multivariable analyses were conducted using Cox proportional hazards and logistic regression models. Statistical analyses were performed using the open-source statistical software R (R Core Team, 2017. R: A Language and Environment for Statistical Computing. R Foundation for Statistical Computing, Vienna, Austria; https://www.R-project.org/ (accessed on 20 April 2026)).

### 2.4. Use of Artificial Intelligence

Artificial intelligence-assisted language editing was used during final manuscript preparation for grammar and stylistic refinement. All scientific content, interpretation, and conclusions were thereafter reverified by the authors (OpenAI (2026) ChatGPT Plus [Large language model]) https://chatgpt.com/plus/ (accessed on 28 April 2026).

## 3. Results

### 3.1. Definition of GBM Five-Year Survivors and Cohorts

In total, 89 GBM five-year survivors were identified and analyzed across two separate cohorts.

### 3.2. The Swedish Cohort

A total of 12,765 patients with diagnoses corresponding to the specified topography and morphology codes were identified from the Swedish Cancer Registry (SCR) and the Swedish Cause of Death Register (SCDR) in Sweden between 1958 and 1999. Of these, 733 patients (5.7%) were initially identified as five-year survivors. Data from the remaining 12,032 non-five-year survivors were anonymized and not reviewed further.

Complete pathology reports and medical records were retrievable for 582 of the 733 five-year survivors (79.4%). Data from 151 patients (20.6%) could not be retrieved, mainly from the earlier decades of the registry period (1958–1969 and 1970–1979). After review of the pathology reports, 497 of the 582 reviewed cases (85.4%) were determined not to represent true GBM diagnoses. The most common alternative diagnoses included anaplastic astrocytoma, WHO grade II astrocytoma, grade III oligoastrocytoma, pilocytic astrocytoma (WHO grade I), grade II oligodendroglioma, and anaplastic oligodendroglioma (WHO grade III).

**Figure 1:**
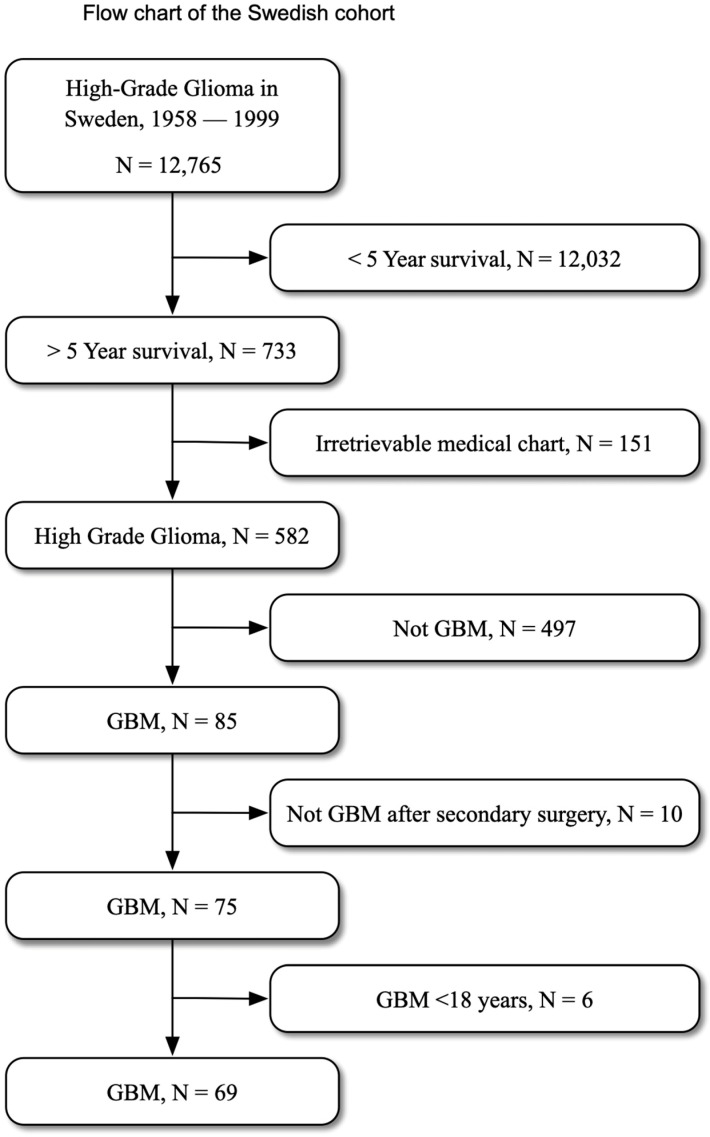
Flow chart of the Swedish cohort.

Eighty-five patients were initially considered to have true GBM; however, in ten cases the diagnosis was revised following secondary surgery and repeat pathological evaluation, most often revealing features consistent with oligodendroglioma. Of the remaining 75 confirmed GBM five-year survivors, six patients were younger than 18 years of age and were therefore excluded from further analysis. In total, 69 adult GBM five-year survivors were identified in the Swedish cohort between 1958 and 1999 (Figure 1).

Hence, 88.1% of the reviewed 582 high-grade glioma (HGG) five-year survivors had other diagnoses than GBM. After stratifying these GBM patients into five-year time periods the number of five-year survivors ranged between 10–21 patients/period (0.3–0.7%). Twenty-nine (0.2%) patients survived more than 10 years, with ten-year survival in the respective time period ranging from 0.1 to 0.3% (Table 1).

Three of the 69 patients belonged to the Southeastern healthcare region during the period 1990–1999 and were therefore transferred to the Southeastern cohort, reducing the number of GBM five-year survivors in the Swedish cohort to 66.

### 3.3. The Southeastern Cohort

**Figure 2:**
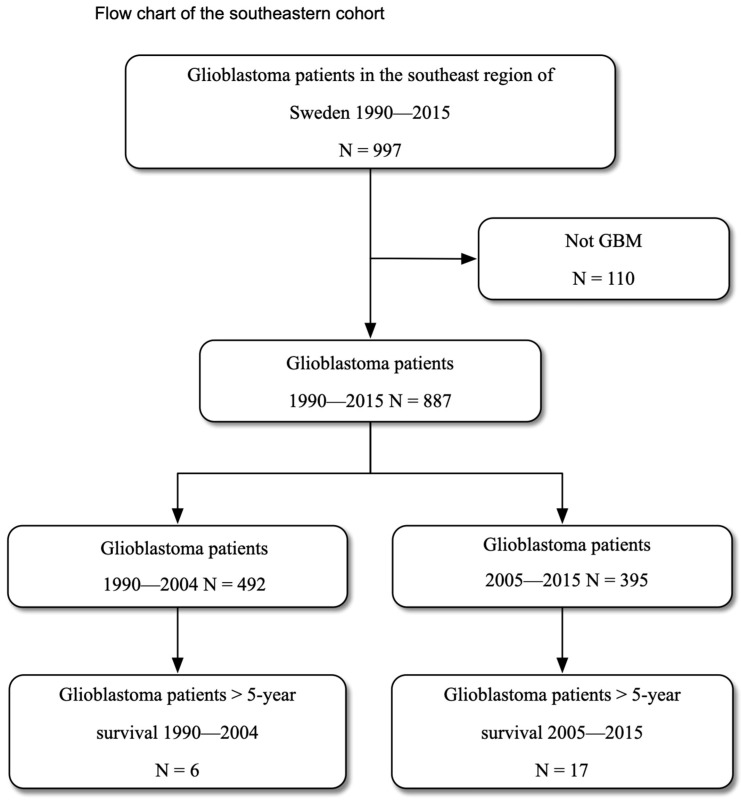
Flow chart of the southeastern cohort.

In the Southeastern region, 997 patients with a registered diagnosis of GBM were identified in the SCR and SBTR between 1990 and 2015. Of these, 38 patients survived longer than five years. A total of 110 patients (11%), including 15 long-term survivors, had been incorrectly registered as GBM and were therefore excluded. In total, 887 histologically verified GBMs and 23 five-year survivors (2.6%) were identified in the Southeastern cohort (Figure 2).

Thus, 15 of the 38 patients (39.5%) would erroneously have been classified as GBM five-year survivors if the diagnoses had not been re-evaluated. There was a significant increase in the proportion of GBM five-year survivors between 1990–1999 and 2010–2015 (*p* = 0.014), between 1990–1999 and 2005–2015 (*p* = 0.014), between 1990–2004 and 2010–2015 (*p* = 0.010), and between 1990–2004 and 2005–2015 (*p* = 0.007) (Table 2). Five-year survival increased progressively from 1.0% in 1990–1999, to 1.7% in 2000–2004, 3.8% in 2005–2009, and 4.7% in 2010–2015 (Table 3). Seven patients (0.8%) survived longer than ten years, with ten-year survival rates ranging from 0.3% to 1.1% across the respective time periods. Notably, no corresponding increase in ten-year survival was observed, in contrast to the significant improvement seen in five-year survival (Table 3).

**Figure 3:**
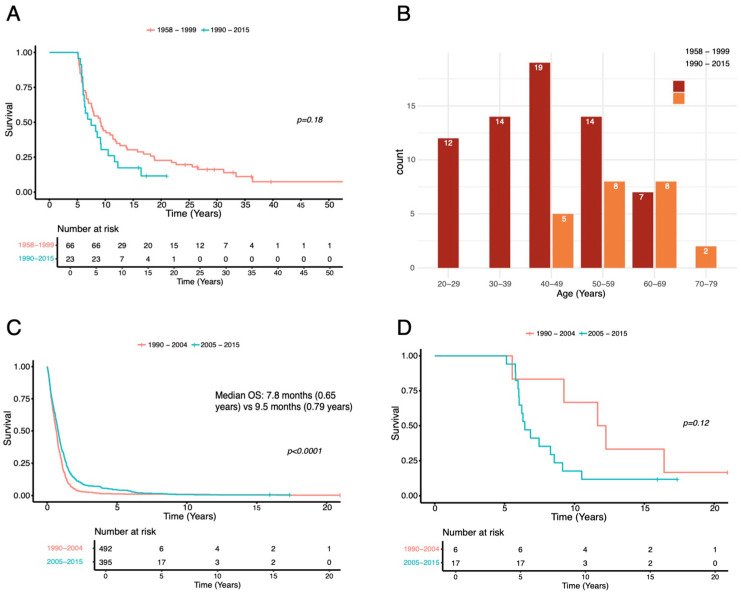
(**A**) Overall long-term survival in the Swedish cohort (1959–1999) and the Southeastern cohort (1990–2015); (**B**) age distribution among the 89 GBM five-year survivors; (**C**) overall survival in the Southeastern cohort during 1990–2004 (pre-temozolomide era) and 2005–2015 (post-temozolomide era); and (**D**) five-year survival in the Southeastern cohort during 1990–2004 (pre-temozolomide era) and 2005–2015 (post-temozolomide era). *p*-values were calculated using log-rank analysis.

While median overall survival (OS) in the entire Southeastern cohort improved significantly from 7.8 months to 9.5 months between the periods 1990–2004 and 2005–2015 (pre- versus post-temozolomide era) (*p* = 0.001) (Figure 3C), no corresponding significant difference was observed among GBM five-year survivors (*p* = 0.12) (Figure 3D). Similarly, there was no significant difference in survival between five-year survivors in the Swedish and Southeastern cohorts (*p* = 0.18) (Figure 3A).

In summary, the proportion of GBM five-year survivors in the Southeastern cohort increased significantly following the introduction of concomitant and adjuvant temozolomide treatment. However, once the five-year survival benchmark had been reached, patients did not survive significantly longer than GBM five-year survivors from the pre-temozolomide era in either cohort. Numerically, survival was rather shorter, although this difference was not statistically significant (Figure 3A).

### 3.4. Incorrect Diagnoses

The proportion of incorrect diagnoses (non-GBMs) among presumed five-year survivors was substantially higher in the Swedish cohort (88.1%) compared with the Southeastern cohort (39.5%), as well as compared with both the 1990–2004 (*p* = 0.003) and 2005–2015 (*p* = 0.001) periods in the Southeastern cohort. Without pathological re-evaluation, the estimated proportions of five- and ten-year survivors in the Southeastern cohort between 1990 and 2015 would have been overestimated to 3.9% and 2.2%, respectively.

### 3.5. Patient and Tumor Characteristics of GBM Five-Year Survivors

Median age at GBM diagnosis was 41 years (range 22–69) in the Swedish cohort and 60 years (range 41–75) in the Southeastern cohort (Table 4, Figure 3B). In the Swedish cohort, 68.2% of patients were younger than 50 years of age, whereas only 21.7% were younger than 50 years in the Southeastern cohort (Table 4).

Sex distribution was equal in the Swedish cohort (50% female). In the Southeastern cohort, there was a slight but non-significant predominance of female patients (56.5%) (Table 4).

Preoperative Karnofsky Performance Status (KPS) was >80 in the majority of patients in the Southeastern cohort (69.6%), whereas this was observed in only 33.3% of patients in the Swedish cohort (Table 4).

MGMT promoter methylation status was not routinely analyzed in patients younger than 65 years before 2015 and was not assessed at all in the Swedish cohort (Table 4). In the Southeastern cohort, three patients (13.0%) had unmethylated MGMT, while two patients (8.7%) had methylated MGMT. MGMT status was unavailable in the remaining 18 patients (78.3%).

Tumors were most commonly located in the frontal and temporal lobes in both cohorts (71.9%), were predominantly superficial in location (60.6%), and were slightly more frequent in the right cerebral hemisphere (56.2%) (Table 4).

### 3.6. Treatment Characteristics

Among GBM five-year survivors in the Swedish cohort, 48.5% underwent GTR, 43.9% PR, and 7.6% biopsy only, compared with 82.6%, 13.0%, and 4.4%, respectively, in the Southeastern cohort. In the latter cohort, eight patients underwent fluorescence-guided surgery and ten patients had postoperative MRI evaluation. Almost all patients in both cohorts received radiotherapy (93.9% and 95.6%, respectively), and the majority were treated with radiation doses exceeding 50 Gy. Chemotherapy was administered to 48.5% of patients in the Swedish cohort, whereas all patients (100%) in the Southeastern cohort received chemotherapy. Of these, 74% were treated with concomitant chemoradiotherapy, 13% received temozolomide monotherapy, and 13% were treated with nitrosourea-based chemotherapy (Table 5). Eighteen patients (27.3%) in the Swedish cohort underwent surgery for tumor recurrence, including two patients who underwent repeat reoperation twice. In the Southeastern cohort, 13 patients (34.8%) underwent one repeat surgical procedure, while one patient underwent three additional operations (Table 5). Histopathological re-evaluation was performed in 39 patients (44%), either following reoperation or because of unexpectedly prolonged survival. In the Southeastern cohort, the majority of patients (56.5%) received temozolomide at recurrence, while 39.1% were treated with lomustine as second-line therapy (Table 5).

### 3.7. Symptoms and Clinical Characteristics

Presenting symptoms (headache, nausea, vomiting, vertigo, neurological deficits, epilepsy, and allergy) as well as selected clinical characteristics (postoperative infection, personal history of other tumors, and family history of tumors) were analyzed in relation to five-year survival using log-rank analysis.

The most common presenting symptoms were neurological deficits (60.7%), headache (55%), and nausea (30.7%). Epilepsy as an initial symptom was present in approximately one-third of the GBM five-year survivors. Absence of vertigo was observed in 71.9% of patients and was significantly associated with five-year survival (*p* < 0.001), as was absence of allergy. However, only one patient had a documented allergy. Postoperative infections, as well as personal or familial histories of other tumors, were observed only in a small minority of patients (approximately 6%) (Table 6).

### 3.8. Outcome Among All Five-Year Survivors

**Figure 4:**
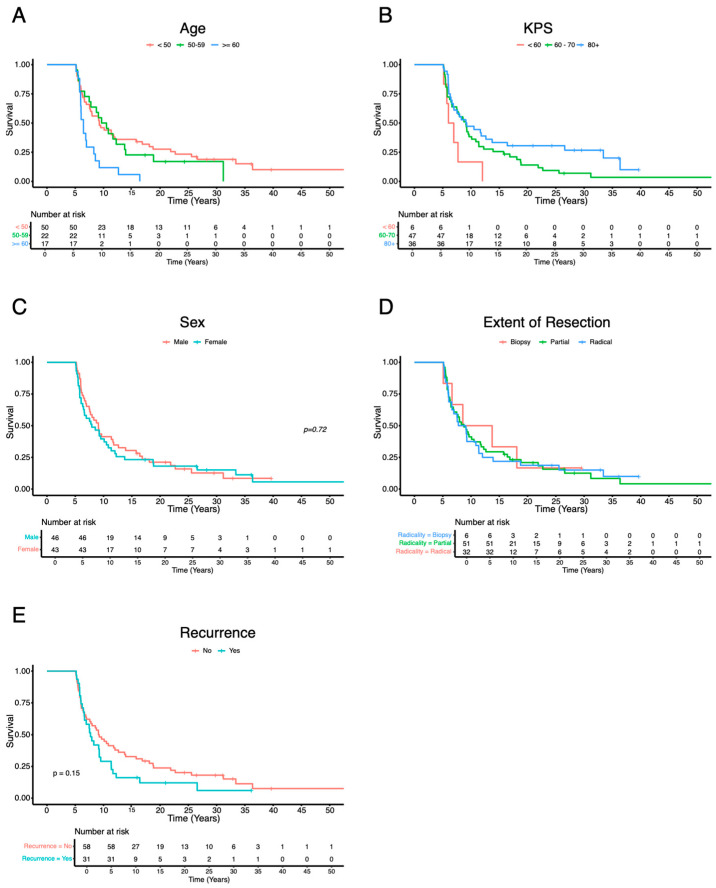
Overall survival in the whole glioblastoma cohort of 89 patients by: (**A**) Age group (Age 18–49 vs. 50–59, *p* = 0.541; Age 18–49 vs. 60–99, *p* = 0.046; “Age 50–59 vs. 60–99; *p* = 0.174); (**B**) Karnofsky Performance Status (KPS < 60 vs. 60–70, *p* = 0.056; KPS < 60 vs. ≥80, *p* = 0.005; “KPS 60–70 vs. ≥80, *p* = 0.022; (**C**) Sex; (**D**) Extent of Resection (Radical vs. Partial, *p* = 0.811; Radical vs. Biopsy, *p* = 0.708; Biopsy vs. Partial, *p* = 0.777; (**E**) Recurrence. *p*-values are from log-rank analysis.

The median overall survival (OS) among all 89 GBM five-year survivors was 8.7 years (95% CI 7.5–10.5) (Table 7). There were no significant differences in long-term survival with respect to sex, extent of resection (GTR, PR, or biopsy), or recurrence versus no recurrence, either in log-rank analyses or in univariate Cox regression analyses (Table 7; Figure 4C–E).

Younger age was significantly associated with longer survival in both the log-rank analysis (*p* = 0.003) and the univariate Cox regression analysis (*p* = 0.001 and *p* = 0.016, respectively) (Table 7; Figure 4A–B). Similarly, a high preoperative Karnofsky Performance Status (KPS) was significantly associated with prolonged survival (*p* = 0.027 and *p* = 0.014, respectively) (Table 7; Figure 4B). Second-line chemotherapy other than temozolomide was also significantly associated with long-term survival in both log-rank analysis (*p* = 0.014) and univariate Cox regression analysis (*p* = 0.015) (Table 7). In the multivariable Cox regression analysis, younger age (<50 years, *p* < 0.001; 50–59 years, *p* = 0.003) remained significantly associated with prolonged survival, whereas lower KPS was significantly inversely associated with five-year survival (KPS 60–70, *p* = 0.038; KPS < 60, *p* = 0.002). All other patient- and treatment-related variables were not significantly associated with survival in either univariate or multivariable analyses (Table 7).

### 3.9. Logistic Regression Analysis of Five-Year Survival in the Southeastern Region

A logistic regression analysis was performed among GBM patients in the southeastern region. In the univariate analysis there was a significant correlation to five-year survival with regard to age (*p* < 0.001), concomitant TMZ (*p* = 0.001), adjuvant TMZ (*p* < 0.001) and reoperation (*p* < 0.001). In the multivariable assessment reoperation (*p* = 0.010) correlated significantly while age was inversely correlated to five-year survival (*p* = 0.002) (Table 8).

### 3.10. Ten-Year Survivors

Thirty-six patients (40.4%) survived for ≥10 years after diagnosis. Of these, eight patients (22.2%) were alive at the time of database closure. Median age at GBM diagnosis among the ten-year survivors was 40 years (range 22–67 years), and the median overall survival was 18 years. Twenty-one patients (58.3%) underwent gross total resection (GTR), 34 patients (94.4%) received radiotherapy, and 26 patients (72.2%) were treated with first-line chemotherapy. Five patients (13.9%) received treatment according to the Stupp regimen, of whom three (8.3%) were still alive at the end of follow-up. Histopathological re-evaluation confirming the GBM diagnosis was performed in 16 cases (44.4%).

Tumor recurrence was identified as the cause of death in eight patients (22.2%). Despite extensive efforts to determine the cause of death, this information remained unavailable in 12 patients (33.3%). In another eight patients (22.2%), death was attributed to causes other than tumor progression. None of these patients had received neuro-oncological treatment prior to death, suggesting a stable disease state without evidence of recurrent tumor activity. One patient who had initially undergone biopsy only died from tumor progression after 18 years of survival. Otherwise, no clear evidence of tumor-related mortality beyond 16 years of overall survival could be identified. In total, 16 patients (44.4%)—comprising eight living patients and eight patients who died from non-tumor-related causes—were interpreted as having achieved complete tumor remission (Table 9).

## 4. Discussion

The year 2026 marks one hundred years since the term “glioblastoma multiforme” was first introduced by Percival Bailey and Harvey Cushing [39]. For nearly 80 years, GBM remained one of the most challenging malignancies in neuro-oncology, with minimal therapeutic progress and persistently poor prognosis [2,40]. Five-year survival was exceedingly rare and historically reported to be approximately 1–2% [16,17]. During the past two decades, however, major paradigm shifts have occurred in the clinical management of GBM. Fluorescence-guided surgery, concomitant radiochemotherapy, and tumor-treating fields (TTF) have all contributed to improved survival, particularly in clinical trial settings, although their impact on long-term survival in population-based cohorts remains less clear and has not been unequivocally demonstrated [8,34,41,42].

In 2016, molecular genetics were incorporated into the histopathological classification of brain tumors, with further refinements introduced in the 2021 WHO classification [43]. However, concerns have been raised that “molecular GBMs” may represent a biologically distinct entity compared with “histological GBMs,” exhibiting more favorable prognoses and longer overall survival [44,45]. This creates a challenge when comparing historical and contemporary cohorts, as evolving diagnostic criteria may lead to discrepancies in GBM classification over time.

The primary aim of the present study was to investigate five- and ten-year survival in GBM over a 57-year period. We demonstrated extremely low rates of long-term survival before 2005, i.e., prior to the introduction of concomitant radiochemotherapy and adjuvant temozolomide treatment. Although the proportion of five-year survivors increased in the post-temozolomide era, patients who reached the five-year benchmark did not survive significantly longer thereafter. This may suggest that the beneficial effect of radiochemotherapy diminishes over time in GBM long-term survivors, providing a substantial palliative effect without clearly achieving cure.

Several clinical variables, including epilepsy, allergy, and postoperative infections, have previously been proposed to influence both median and long-term survival in GBM patients [46,47]. In the present study, only vertigo was associated with shorter survival among five-year survivors. Interestingly, treatment with chemotherapy other than temozolomide was significantly associated with long-term survival. However, interpretation of this finding is difficult, as 87% of patients in the Southeastern cohort received temozolomide as first-line therapy, whereas none of the patients in the historical Swedish cohort did so.

In the Southeastern cohort, the proportion of five-year survivors increased significantly during the most recent time periods, coinciding with the introduction of the Stupp regimen in 2005. Several previous reports have documented progressive improvements in population-based GBM overall survival following the implementation of temozolomide [30,31,32], particularly for one-, two-, and three-year survival, although not consistently for five-year survival [33]. Some studies have also suggested a plateau effect after approximately 2011 [48]. In contrast, our data demonstrated a continued increase in five-year survival after 2011 in the Southeastern region, peaking during the 2010–2015 period.

In the logistic regression analysis of five-year survival in the Southeastern cohort, significant associations were observed for age, radiotherapy, concomitant temozolomide, adjuvant temozolomide, reoperation, and second-line chemotherapy, whereas extent of resection, sex, and second-line radiotherapy were not significant in univariate analyses. Notably, extent of resection did not appear to play a decisive role in achieving five-year survival in this cohort. However, none of the radiochemotherapy-related variables remained significant in multivariable analyses, which may partly reflect the relatively small number of patients receiving full concomitant radiochemotherapy.

Similarly, in the multivariable Cox regression analysis of all 89 five-year survivors, only age and preoperative Karnofsky Performance Status (KPS) were significantly associated with prolonged survival, whereas all treatment-related factors were non-significant.

In contrast to five-year survival, no corresponding improvement was observed for ten-year survival. This may indicate exhaustion of treatment efficacy over time or, speculatively, treatment-induced biological mechanisms contributing to more aggressive tumor behavior [49]. In oncology, five-year survival is generally considered a marker of sustained palliation, whereas ten-year survival may approximate, though not necessarily equal, cure [50].

We identified 36 ten-year survivors, which, to our knowledge, represents one of the largest clinically verified series of GBM ten-year survivors reported to date. Apart from a few smaller studies [51,52], clinical investigations of very long-term survivors (VLTS) remain scarce [21,22,53,54,55]. In a study by Marton et al., gross total resection was achieved in five of seven VLTS patients and MGMT methylation was present in six [53]. In our cohort, evidence of tumor recurrence causing death was observed in eight patients with overall survival ranging from 10 to 18 years after diagnosis, including one patient who had undergone biopsy only. Beyond 18 years of survival, however, we found no clear evidence of tumor-related mortality. This observation may tentatively suggest the existence of a time threshold beyond which complete remission or cure becomes possible in GBM, although this interpretation should be approached cautiously and warrants further investigation.

Our observed ten-year survival rate of approximately 1% is consistent with previous meta-analytic data [21], although substantially lower than the 4.4% reported for all age groups in the most recent CBTRUS report [10].

A notable finding was the high proportion of incorrect diagnoses among presumed five-year survivors extracted from the registries. In particular, 15 long-term survivors had been incorrectly registered as GBM. The reason for these inaccuracies is unclear but may reflect diagnostic or registration errors. Importantly, without careful re-evaluation, these cases would have substantially inflated both five- and ten-year survival estimates, underscoring the importance of rigorous validation of registry data, particularly when analyzing long-term survival outliers.

Both five- and ten-year survival rates in the post-temozolomide era were substantially lower in our population-based cohorts than those reported in the most recent CBTRUS analysis [10]. This discrepancy may partly reflect inclusion of pediatric patients in CBTRUS data, misclassification of diagnoses in anonymized datasets, or other forms of selection bias. Interestingly, if incorrect diagnoses had not been excluded in our study, the estimated five-year survival rates would have been closer to previously published reports that did not include pathological re-verification.

The molecular factors associated with GBM long-term survival have been investigated extensively, although interpretation remains difficult because of varying definitions of LTS [23,24,25,26,27]. We suggest that LTS in GBM should be defined as survival beyond five years. Apart from hypermethylation phenotypes and MGMT promoter methylation [26,27], no universally predictive prognostic factor has yet been identified [29]. Moreover, while some studies have demonstrated a strong association between MGMT methylation and LTS [24,26], others have failed to confirm this relationship [29,56]. MGMT status was not routinely assessed before 2015, and data were available for only five patients in our cohort. Interestingly, three of these patients had unmethylated MGMT, indicating that MGMT methylation is not an absolute prerequisite for prolonged survival.

### Limitations and Strengths

This study has several limitations. First, the retrospective design may have resulted in incomplete or inaccurate data collection. Furthermore, the exact number of GBM patients surviving less than five years in the historical Swedish cohort was unavailable, precluding precise calculation of true five-year survival rates. Nevertheless, applying the proportion of incorrect diagnoses observed in short-term survivors from the Southeastern cohort to the historical Swedish cohort would not substantially alter the estimated survival rates.

Second, all GBM diagnoses up to 2015 were based solely on histopathological criteria, which may both overestimate and underestimate long-term survival according to the most recent WHO classifications. This could potentially include IDH-mutant grade 4 astrocytomas while excluding “molecular GBMs” with lower-grade histological features. However, all secondary GBMs, which are more likely to harbor IDH mutations, were excluded from this study.

Importantly, this study was specifically designed to evaluate ten-year survival, which necessitated a patient inclusion cutoff on 31 December 2015, with survival follow-up extending until 31 December 2025. Consequently, patients were diagnosed before the introduction of the 2016 molecular WHO classification, limiting the possibility of comprehensive molecular analyses in this study. Similarly, MGMT methylation status was only available in a minority of patients.

Third, no centralized pathology review was performed. Histopathological interpretation is inherently subjective and may contribute to diagnostic inaccuracies [57]. For example, the markedly younger age at diagnosis in the historical Swedish cohort compared with the Southeastern cohort raises concerns regarding diagnostic consistency, particularly in earlier decades. Nevertheless, histopathological re-evaluation was performed in 44% of cases, confirming the GBM diagnosis.

The major strengths of this study include the extensive verification of all retrievable pathology reports and medical records among five-year survivors in the Swedish cohort and all GBM patients in the Southeastern cohort, thereby correcting erroneous registry data. In addition, patient registration in the SBTR from the Southeastern region has been reported to approach 100%, supporting the robustness and reliability of the population-based data.

## 5. Conclusions

This study represents one of the largest registry-verified, population-based analyses of histologically diagnosed GBM five- and ten-year survivors. Five-year survival increased significantly following the introduction of the Stupp regimen into clinical practice in 2005, suggesting—but not proving—a causal relationship with modern neuro-oncological treatment strategies. From a population-based perspective, five-year survival appears increasingly attainable, whereas ten-year survival remains exceedingly rare. We found no evidence of tumor recurrence or tumor-related mortality beyond 18 years of overall survival. Careful verification of diagnoses in registry-based survival analyses is essential for accurately assessing the long-term impact of therapeutic advances in GBM.

## Figures and Tables

**Table 1:** Distribution of total HGG cases, HGG 5-year survivors and verified five and ten-year GBM survivors in Sweden 1958–1999.

Time Cohorts	Total HGG, no	HGG > 5 yr no (%)	GBM > 5 yr, no (%)	GBM > 10 yr, no (%)
1958–1969	3291	188 (5.7)	10 (0.3)	4 (0.1)
1970–1979	3119	149 (4.7)	19 (0.6)	6 (0.2)
1980–1989	3193	187 (5.8)	21 (0.7)	10 (0.3)
1990–1999	3162	209 (6.6)	19 (0.6)	9 (0.3)
1958–1999	12,765	733 (5.7)	69 (0.5)	29 (0.2)

**Table 2:** GBM vs. GBM 5-year survival in different time periods in the southeastern region of Sweden during 1990–2015. Chi Square Analysis.

Time Period	GBM 5-Year Survivors	GBM Patients	Time Period	GBM 5-Year Survivors	GBM Patients	*p*-Value
1990–1999	3	312	2005–2009	7	176	0.061
1990–1999	3	312	2010–2015	10	202	0.014 *
1990–1999	3	312	2005–2015	17	378	0.014 *
1990–1999	3	312	2000–2004	3	174	0.770
1990–2004	6	486	2005–2015	17	378	0.007 *
1990–2004	6	486	2005–2009	7	176	0.061
1990–2004	6	486	2010–2015	10	202	0.010 *
2000–2004	3	174	2005–2009	7	176	0.363
2000–2004	3	174	2010–2015	10	202	0.171
2000–2004	3	174	2005–2015	17	378	0.185

* significance level.

**Table 3:** Distribution of verified GBM cases with five- and ten-year GBM survivors in the southeastern region of Sweden 1990–2015.

Time Cohorts	Total GBM, No	GBM > 5 yr, no (%)	GBM > 10 yr, no (%)
1990–1999	315	3 (1.0)	1 (0.3)
2000–2004	177	3 (1.7)	3 (1.7)
2005–2009	183	7 (3.8)	2 (1.1)
2010–2015	212	10 (4.7)	1 (0.5)
**1990–2015**	**887**	**23 (2.6)**	**7 (0.8)**

**Table 4:** Patient characteristics—GBM 5-year survivors in Sweden 1958–1999 * (*n* = 66) and in the southeastern region of Sweden 1990–2015 (*n* = 23).

		GBM > 5 yr 1958–1999 in Sweden * (n = 66) n (%)	GBM > 5 yr 1990–2015 in Southeast Sweden (n = 23) n (%)
Age	Median	41	60
	Range	22–69	41–75
Age groups	<50	45 (68.2)	5 (21.7)
	50–59	13 (19.7)	8 (34.8)
	>60	8 (12.1)	10 (43.5)
Sex	Male	33 (50.0)	10 (43.5)
	Female	33 (50.0)	13 (56.5)
KPS preop	80–100	22 (33.3)	16 (69.6)
	60–70	38 (57.6)	4 (17.4)
	<60	6 (9.1)	3 (13.0)
MGMT (%)	Unmethylated	0 (0)	3 (13.0)
	Methylated	0 (0)	2 (8.7)
	Missing	66 (100)	18 (78.3)
Tumor localization	Frontal	37 (56.1)	7 (30.4)
	Temporal	13 (19.7)	7 (30.4)
	Parietal	6 (9.1)	4 (17.4)
	Occipital	6 (9.1)	4 (17.4)
	Central	4 (6.0)	1 (4.3)
	Superficial	39 (59.1)	15 (65.2)
	Deep	27 (40.9)	8 (34.8)
	Right side	40 (60.6)	10 (43.5)
	Left side	26 (39.4)	13 (56.5)
Survival	Range	5–55 years	5–19 years
	Median OS	9.2 years	7.5 years
	Mean OS	13.6 years	8.7 years
	Events (death)	61 (92.4)	20 (87.0)
	Alive at last follow up	5 (7.6)	3 (13.0)

* Except the southeastern region of Sweden 1990–1999.

**Table 5:** Treatment characteristics—GBM 5-year survivors in Sweden 1958–1999 * (*n* = 66) and the southeastern region of Sweden 1990–2015 (*n* = 23). Chi Square analysis.

		GBM > 5 yr 1958–1999 in Sweden * (n = 66) n (%)	GBM > 5 yr 1990–2015 in Southeast Sweden (n = 23) n (%)	*p*-Value
Extent of surgery	GTR	32 (48.5)	19 (82.6)	0.009 *
	Partial resection	29 (43.9)	3 (13.0)	0.016 *
	Biopsy	5 (7.6)	1 (4.4)	0.961
Radiotherapy as first-line treatment	Yes	62 (93.9)	22 (95.6)	1
	No	4 (6.0)	1 (4.3)	1
	50–60 Gy	32 (48.5)	20 (87.0)	0.003 *
	40–50 Gy	3 (4.5)	0 (0)	0.712
	<40 Gy	0 (0)	2 (8.7)	0.579
	Missing data	27 (40.9)	0 (0)	0.003 *
Chemotherapy as first line treatment	Yes	28 (42.4)	23 (100)	0.001 *
	No	38 (57.6)	0 (0)	0.001 *
	TMZ concomitant	0 (0)	17 (74.0)	0.001 *
	TMZ monotherapy	0 (0)	3 (13.0)	0.021 *
	Nitrosurea	28 (42.4)	3 (13.0)	0.176
	No treatment	38 (57.6)	0 (0)	0.001 *
Surgery at recurrence	Yes	18 (27.3)	13 (56.5)	0.022 *
	No	48 (72.7)	10 (43.5)	0.022 *
Number of recurrences	0	50 (75.7)	8 (34.8)	0.001 *
	1	18 (27.3)	13 (56.5)	0.022 *
	2	2 (3.0)	1 (4.4)	1
	3	0 (0)	1 (4.4)	0.279
Radiotherapy at recurrence	yes	0 (0)	3 (13.0)	0.021 *
	no	66 (100)	20 (87.0)	0.021 *
TMZ at recurrence	yes	3 (4.5)	13 (56.5)	0.001 *
	no	63 (95.5)	10 (43.5)	0.001 *
Nitrosourea at recurrence	yes	8 (12.1)	9 (39.1)	0.011 *
	no	58 (87.9)	14 (60.9)	0.011 *
Bevacizumab at recurrence	yes	0 (0)	1 (4.4)	0.579
	no	66 (100)	22 (95.6)	0.579

* Except the southeastern region of Sweden 1990–1999.

**Table 6:** Symptoms and traits in all 89 GBM 5-year survivors and Overall Survival (days). *n* = 89. Chi Square analysis.

Symptoms and Traits	Yes (%)	Median OS	No (%)	Median OS *	*p*-Value
Headache	49 (55.0)	3381	40 (45.0)	2633	0.503
Nausea	32 (36.0)	3158	57 (64.0)	3321	0.875
Vomit	27 (30.3)	3326	62 (69.7)	3075	0.592
Vertigo	25 (28.1)	2549	64 (71.9)	3379	0.002 *
Neurological deficits	54 (60.7)	3075	35 (39.3)	3377	0.053
Epilepsy	30 (33.7)	2820	59 (66.3)	3326	0.568
Allergy	1 (1.1)	2025	88 (98.9)	3257	0.023 *
Postop infection	5 (5.6)	3321	84 (94.4)	3158	0.504
Other Tumor	6 (6.7)	3767	83 (93.3)	3125	0.733
Heredity tumor	5 (5.6)	3381	84 (94.4)	3075	0.746

* significance level.

**Table 7:** Log-rank, Univariate and Multivariate Cox regression analysis with regard to death. *n* = 89.

							Cox Regression Analysis			
				Log-Rank Analysis			Univariate		Multivariable	
		n	Events	Median OS (Years)	95% CI	*p*-Value	HR * (95% CI)	*p*-Value	HR * (95% CI)	*p*-Value
All patients		89	78	8.7	7.5–10.5		-	-		
Age groups	<50	50	42	9.2	7.6–16.9		0.37 (1.22–1.96)	0.001 *	0.31(0.15–0.61)	<0.001
	50–59	22	19	10.1	7.8–13.9		0.44 (1.25–2.36)	0.016 *	0.35(0.17–0.70)	0.003
	>60	17	17	6.4	6.0–8.6	0.003 *	1	ref		
Sex	male	43	38	7.9	6.4–11.4	0.713	1	ref		
	female	46	40	9.1	7.6–12.6		0.920 (1.80–4.21)	0.714		
KPS preop	80–100	36	28	9.2	6.9–16.4	0.027 *	1	ref		
	60–70	47	44	9.1	7.5–11.4		1.52 (2.56–11.69)	0.087	1.7 (1.03–2.81)	0.038
	<60	6	6	6.5	NA		3.08(3.49–2007.98)	0.014 *	4.49(1.70–11.85)	0.002
Extent of surgery	GTR	32	28	8.4	6.6–11.4	0.942	1	ref		
	PR	51	45	8.7	7.0–11.8		0.99 (1.85–4.90)	0.966		NS
	Biopsy	6	5	11.1	NA		0.85 (1.39–9.07)	0.736		NS
Radiotherapy	yes	82	71	9.1	7.6–11.4		0.66 (1.30–5.2)	0.375		
	no	5	5	5.8	5.8–NA	0.372	1	ref		
Concomitant TMZ	Yes	18	15	7.1	6.3–11.7		1.32 (2.11–10.56)	0.339		
	No	71	63	9.4	7.7–11.8	0.337	1	ref		
Treatment with other chemo	yes	34	27	11.7	9.1–18.8		0.55 (1.41–2.44)	0.015 *		NS
	No	55	51	7.6	6.4–9.2	0.014 *	1	ref		
Recurrence	Yes	31	28	7.7	6.6–11.4		1.41 (2.41–9.61)	0.153		
	No	58	50	9.2	7.6–13.7	0.148	1	ref		
Number of recurrences	0	58	50	9.2	7.6–13.7	0.136	1	ref		
	1	25	22	7.6	6.6–11.4		1.42 (2.35–10.75)	0.174		
	2	5	5	9.5	8.2–NA		1.20 (1.61–20.54)	0.698		
	3	1	1	5.8	NA		6.54 (2.34–6.66)	0.07		

* HR = Hazard Ratio. NA: Not Applicaple; NS: Not Significant.

**Table 8:** Univariate and multivariable logistic regression of five-year survival as outcome in the southeastern region (*n* = 887).

	Logistic Regression Analysis			
	Univariate		Multivariable	
	OR * (95% CI)	*p*-Value	OR * (95% CI)	*p*-Value
**Age**	0.93 (0.90–0.94)	<0.001	0.31(0.15– 0.61)	0.002
**Gender**	1.35 (0.75–2.44)	0.31		NS
**Radiation**	4.76 (1.67–19.99)	0.01		NS
**Concomitant Tmz**	2.75 (1.47–5.02)	0.001		NS
**Adjuvant Tmz**	4.26 (1.91–10.83)	<0.001		NS
**GTR + PR**	0.8 (0.4–1.70)	0.54		NS
**Reoperation**	5.72 (2.83–11.90)	<0.001	1.7 (1.03–2.81)	0.010
**Second line chemo**	3.53 (0.81–10.93)	0.049		NS
**Second line radiotherapy**	0.67 (0.09–3.47)	0.66		NS

* OR = Odds Ratio. NS: Not Significant.

**Table 9:** Patient and treatment characteristics in ≥ GBM ten-year survivors. *n* = 36.

No.	Sex	OS—Years	Age at Diagnosis	Year of Diagnosis	K-PS	Resection	Recurrence	Radiation (Gy)	Chemotherapy	Re-Evaluation	Life Status	Cause of Death
1	M	10	50	1963	60	PR	0	None	None	No	dead	unknown
2	M	10	34	1986	70	GTR	0	56	NOC	no	dead	unknown
3	M	10	52	2011	100	GTR	0	58	RCT + TMZ	no	dead	tumor
4	F	11	60	2004	80	GTR	1	56	RCT + TMZ + CCNU	yes	dead	tumor
5	F	11	29	1991	90	GTR	0	56	None	no	dead	tumor
6	F	11	38	1980	70	PR	1	52	CCNU	yes	dead	tumor
7	M	11	49	1981	70	PR	2	50	none	yes	dead	tumor
8	M	12	52	1999	70	GTR	1	56	CCNU + TMZ	yes	dead	tumor
9	M	12	38	1986	50	PR	0	56	NOC	no	dead	unknown
10	F	12	60	1999	80	GTR	1	56	CCNU	yes	dead	other
11	F	13	52	1976	70	PR	0	56	CYC	no	dead	other
12	M	13	50	1969	90	Biopsy	0	50	none	no	dead	unknown
13	F	15	38	1987	70	GTR	0	56	BCNU	no	dead	unknown
14	F	16	45	2009	80	GTR	1	60	RCT + TMZ	yes	alive	-
15	F	16	25	1975	70	GTR	0	50	Vindesine	yes	dead	tumor
16	F	16	67	2000	80	GTR	1	56	TMZ	yes	dead	other
17	M	17	51	2007	80	GTR	0	60	RCT + TMZ	no	alive	-
18	M	18	58	1974	70	GTR	0	50	none	no	dead	other
19	F	18	41	1991	70	PR	0	56	NOC	no	dead	unknown
20	M	18	40	1998	70	Biopsy	0	56	CCNU	no	dead	tumor
21	F	21	53	2004	60	GTR	1	60	RCT + TMZ	yes	alive	-
22	F	21	36	1984	60	GTR	0	56	CCNU	yes	dead	other
23	F	22	33	1972	60	GTR	0	50	none	no	dead	unknown
24	F	25	46	1993	70	PR	0	56	none	no	dead	unknown
25	F	26	56	1999	80	PR	0	56	CCNU	no	dead	other
26	M	26	28	1980	80	GTR	1	56	BCNU	yes	dead	unknown
27	M	27	35	1998	70	GTR	0	56	CCNU	yes	alive	-
28	M	30	24	1995	80	GTR	0	56	CCNU	yes	alive	-
29	F	31	54	1966	60	GTR	0	none	none	no	dead	unknown
30	F	31	22	1994	90	Biopsy	0	56	NOC	no	alive	-
31	M	33	23	1977	90	PR	0	50	CCNU	no	dead	unknown
32	F	34	35	1991	80	PR	0	56	CCNU	yes	dead	other
33	M	36	39	1967	100	GTR	0	62	none	no	dead	unknown
34	M	37	40	1987	80	PR	1	56	BCNU	yes	dead	other
35	F	41	41	1984	80	PR	0	56	CCNU	yes	Alive	-
36	M	55	26	1970	70	GTR	0	50	none	no	alive	-

## Data Availability

Patient data was, after ethical approval, as stated above, obtained from the Swedish Cancer Registry (SCR), the Swedish Cause of Death Register (SCDR) and the Swedish Brain Tumor Registry (SBTR). All patient data (medical charts, pathology reports, radiology pictures) was stored in closed repositories and anonymized databases (Excel and Redcap) and has been coded and hence anonymized to everyone except the authors. Anonymized patient data can be shared upon request.

## Abbreviations

The following abbreviations are used in this manuscript:

GBM	Glioblastoma Multiforme
SCR	Swedish Cancer Registry
SCDR	Swedish cause of Death Register
SBTR	Swedish Brain Tumor Registry
STS	Short-Term Survival
LTS	Long-Term Survival
VLTS	Very Long-Term Survival
OS	Overall survival
TMZ	Temozolomide
CCNU	Lomustine
BCNU	Carmustine
NOC	N-Nitroso-Compounds
CYC	Cyclophosphamide
RCT	Radiochemotherapy
IDH	Isocitrate Dehydrogenase
MGMT	O6-methylguanine-DNA methyltransferase
CBTRUS	Central Brain Tumor Registry of the United States
ICD	International Classification of Diseases
GTR	Gross Total Resection
PR	Partial Resection
KPS	Karnofsky Performance Status
LGG	Low Grade Glioma
HGG	High Grade Glioma
WHO	World Health Organization
MRI	Magnetic Resonance Imaging
HR	Hazard ratio
OR	Odds Ratio
NA	Not Applicaple
NS	Not Significant
